# Aspects of Dietary Diversity Changes across Adulthood in Racially Diverse Adults

**DOI:** 10.3390/nu12082455

**Published:** 2020-08-15

**Authors:** Rita Rawal, Marie Fanelli Kuczmarski, Nancy Cotugna, Benjamin C. Brewer, May A. Beydoun, Virginia C. Hughes, Alan B. Zonderman, Michele K. Evans

**Affiliations:** 1 Department of Behavioral Health and Nutrition, University of Delaware, 021 CSB, 26N College Ave, Newark, DE 19716, USA; mfk@udel.edu (M.F.K.); ncotugna@udel.edu (N.C.); 2 College of Health Sciences, University of Delaware, STAR, Newark, DE 19716, USA; bcbrewer@udel.edu; 3 Laboratory of Epidemiology and Population Sciences, National Institute on Aging, NIH, 251 Bayview Blvd. Suite 100, Baltimore, MD 21224, USA; baydounm@mail.nih.gov (M.A.B.); zondermana@gmail.com (A.B.Z.); evansm@grc.nia.nih.gov (M.K.E.); 4 Department of Medical and Molecular Sciences, University of Delaware, 303A Willard Hall, Newark, DE 19716, USA; vhughes@udel.edu

**Keywords:** dietary diversity, count, evenness, dissimilarity, African–American, adults

## Abstract

Knowledge of various aspects of dietary diversity (DD)—an essential healthful dietary component—across adulthood is limited. This study examined three DD aspects over time in racially diverse adults. Participants were from the National Institute on Aging, Healthy Aging in Neighborhoods of Diversity across the Life Span study. DD measures were calculated at baseline (*N* = 2177), and first and second examination follow-ups (*N* = 2140 and *N* = 2066, respectively) using two 24-h recalls. The count was based on the consumption of ≥50% of an equivalent from 21 food groups. Evenness was derived using the Berry-Index adjusted by the food’s health value; dissimilarity, by Mahalanobis Distance. Mixed-effects linear regression models were conducted to test changes in DD across adulthood, adjusting for sex, race, poverty status and education as fixed effects, and adjusting for smoking, age and energy as time-dependent variables. Only dissimilarity showed significant interactions of time × race (*p* = 0.0005), and time × poverty status (*p* = 0.0325), indicating a slower rate of increase over time in dissimilarity scores among Whites compared with African–Americans and those with income >125% poverty versus <125% poverty. A significant interaction between time×energy (*p* < 0.0001) was noted for both evenness and dissimilarity scores. To our knowledge, this is the first study to document the differential change in dissimilarity scores by race and income over time.

## 1. Introduction

Dietary Diversity (DD) is a universally recognized key component of a healthful diet and is considered to reflect variety [1]. There is no standard definition of DD among researchers and the definition varies with the purpose of the study and the outcome of interest. In simple terms, DD is defined as eating varieties of distinct and wholesome foods that promote nutrient adequacy, high dietary quality, and the maintenance of optimal health [1,2,3,4,5,6,7,8,9,10].

Increased variety within food groups has also been recommended to improve public health in the United States and worldwide [11,12,13]. Dietary variety is one of the important components incorporated in the 2015–2020 Dietary Guidelines for Americans with the recommendation for not only including a variety of nutrient-dense foods across but also within food groups, especially vegetables and protein-rich foods [13]. Additionally, DD also helps to promote biodiversity and sustainability [14] along with reducing the risk for various diet-related chronic conditions, such as metabolic syndrome [15], obesity [16] and cardiovascular risk [17,18].

Various approaches have been used by researchers to calculate DD. The most widely used and traditional method uses counts of different foods or food groups consumed over a specific period [19]. However, the use of food and food group classification systems, as well as the length of the reference period, varies across the studies [19]. The limitations of count-based measures include the lack of distinguishing between healthful and unhealthful products, the distribution of individual food quantities, and the inability to capture differences in specific nutrient contents. Recently, to overcome the limitations of the count-based approach, other alternative methods, such as evenness [20,21,22,23] (balance in the distribution of calories across individual foods) and dissimilarity [20] (difference in food consumption with specific health-related attributes) have also been used. These approaches have been adopted from diversity science in ecological and economic systems.

Age-related changes in DD have been reported by Otsuka and colleagues [24]. The authors examined the change in DD among 922 men and 879 women using 12-year longitudinal data from a community-based study. The DD was calculated using the Quantitative Index for Dietary Diversity based on the proportion of foods that contribute to total energy, or the amount of foods and the number of food groups. The results revealed an age-related decline in DD among both sexes, with a significant inverse association noted among older women (63-year-old slope = −0.00033/year, *p* = 0.03; 79-year-old slope = −0.00092/year, *p* < 0.001) [24].

Food variety reflects dietary quality over time [7]. A study by Kuczmarski and colleagues [25] found that dietary quality, measured by mean adequacy ratio, declined with age among Healthy Aging in a Neighborhood of Diversity across the Life Span (HANDLS) study participants. The HANDLS study was designed to explore the independent and/or synergistic effects of race and socioeconomic status on health disparities observed in African–Americans and Whites residing in an urban community. Health disparities adversely affect groups of people who have systematically experienced greater obstacles to health based on factors such as their race or ethnic group, socioeconomic status, sex, age, mental health, physical disability, and gender identity [26]. Previous cross-sectional analyses by Kuczmarski and colleagues [27] revealed the positive association of count and negative association of dissimilarity with 10-year atherosclerotic cardiovascular risk among HANDLS study participants. Few studies have collectively used all three methods—count, evenness, and dissimilarity—to assess DD [20,27]. These studies did not explore change in DD with age. Therefore, the purpose of this study was to examine the change in DD using count, evenness, and dissimilarity in socioeconomically and racially diverse adults from multiple study visits of the HANDLS study. We hypothesize that DD will lessen over time among the HANDLS study participants.

## 2. Methods

### 2.1. Background on Healthy Aging in Neighborhoods of Diversity across the Life Span (HANDLS) Study

The HANDLS survey sample is a fixed cohort consisting of African–American (AA) and White adults from 13 neighborhoods in the city of Baltimore. The baseline sample consisted of 3720 AA and White adults, aged 30–64 years. The study design was a representative factorial cross of four factors: age (30–64 years), sex (male and female), race (AA and White), and income (self-reported household income dichotomized into <125% and >125% of the 2004 Health and Human Services poverty guidelines) [28]. A detailed description of the study can be found elsewhere [29]. The three completed examination waves used for this study include baseline wave (visit (v)1: 2004–2009), the first follow-up examination (v2: 2009–2013), and the second follow-up examination (v3: 2013–2017).

Inclusion in the study required participants aged 30 to 64 years at baseline, the ability to provide informed consent, and possession of valid picture identification. Participants who were pregnant at the time of entry, had diagnoses of AIDS, or were within 6 months of active treatment of cancer were excluded from the study. The study protocol was approved by the human investigation review boards at MedStar Research Institute, the National Institutes of Environmental Health Sciences, National Institutes of Health, and the University of Delaware. All participants provided written informed consent and were compensated monetarily.

Data collection for the initial HANDLS examination occurred in 2 phases [30]. Phase one consisted of a household survey conducted in participants’ homes including the first 24-h dietary recall and a structured interview regarding participants’ health statuses and personal and demographic characteristics. The second phase was conducted in mobile research vehicles (MRV) by trained staff and included a medical history, a second 24-h dietary recall, physical examination, handgrip strength and blood tests, cognitive measures, body composition and bone mineral density evaluation by dual-energy X-ray absorptiometry and standardized anthropomorphic measurements, including height and weight. For v2 and v3, examination data were collected on the MRV, including the first 24-h dietary recalls. The second 24-h dietary recalls were completed by telephone by trained interviewers. The study protocols at each wave along with a complete listing of all the variables by a wave can be found elsewhere [31]. The HANDLS study is registered with ClinicalTrials.gov and the details can be found elsewhere [32].

### 2.2. Study Sample

**Figure 1:**
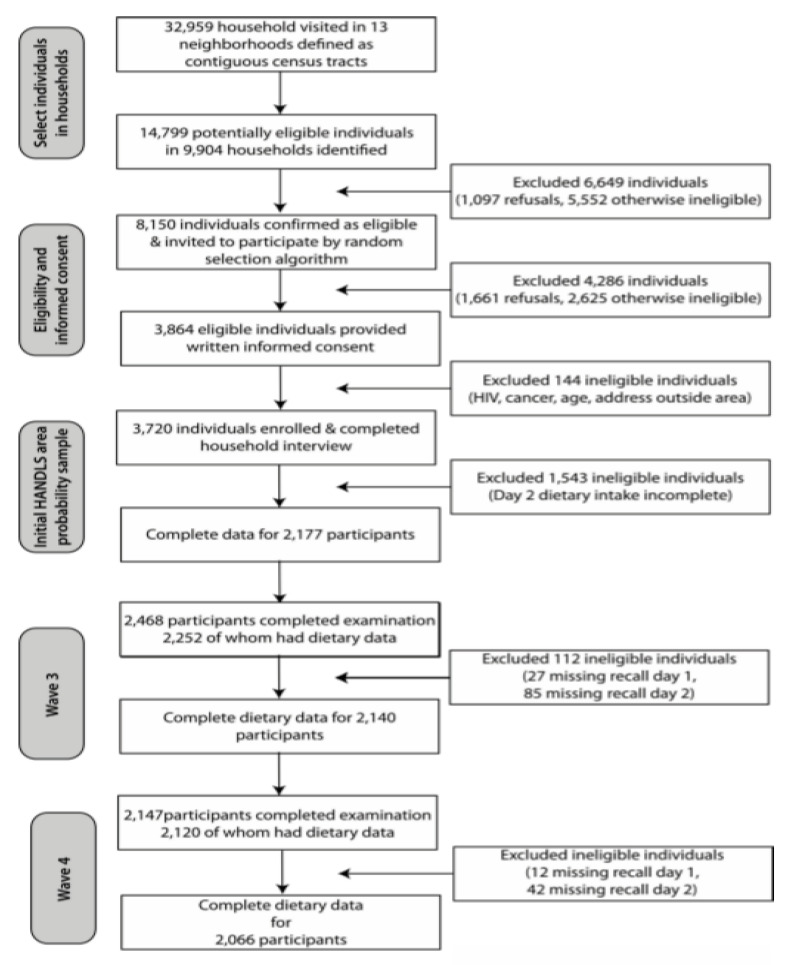
Participants completing two dietary recalls at each of the Healthy Aging in Neighborhood of Diversity across the Life Span study visits.

Of the initial HANDLS study sample, 2177 AA and White adults completed two 24-h dietary recalls at v1, 2140 at v2 and 2066 at v3, respectively (see Figure 1). For this study, only participants who completed two 24-h recalls at any visit were included, with an average number of 1.8 observations per participant.

### 2.3. Dietary Intake Assessment

Two 24-h dietary recalls, approximately 4–10 days apart were conducted by trained interviewers using the validated United States Department of Agriculture (USDA) Automated Multiple Pass Method (AMPM) [33]. There are five steps in the AMPM methods that were used to provide cues and prompts through recall for all foods and drinks consumed throughout the previous day. A detailed description of these steps can be found elsewhere [34]. Household measurement aids, such as measuring cups and spoons, a ruler, and an illustrated Food Model Booklet, were used by participants to assist them in estimating the accurate portion size of foods and beverages consumed. The collected food intake data were coded with unique food codes using the Food and Nutrient Database for Dietary Studies (FNDDS), 3.0 (2005–2006) for v1, FNDDS, 5.0 (2009–2010) for v2, and FNDDS, 2013–2014 for v3 and Survey Net Coding software [35,36].

### 2.4. Dietary Diversity Measurements

Dietary diversity was estimated using count, evenness of food intake distribution and dissimilarity of food items consumed to incorporate various aspects of DD. The count score was based on the consumption of ≥ half cup—or ounce—equivalent of food as defined in the Dietary Guidelines for Americans, 2015–2020 [13]. Foods consumed were assigned to groups using codes defined in the USDA Food Patterns Equivalents Database (FPED), 2005–2006 for v1, FPED, 2009–2010 for v2, and FPED, 2013–2014 for v3 [37,38,39]. To avoid counting duplicate foods, the quantity of food items with the same food codes consumed within 24 h was aggregated for each day before assigning equivalents. A total of 21 food groups considered healthful were used. The detailed description of the count score can be found elsewhere [27]. The final DD count score was derived by the total number of food groups consumed divided by 21. The theoretical score can range between 0 and 1. Lower scores suggest that fewer food groups were consumed, and larger scores indicate that a larger number of food groups were consumed.

The evenness scores were derived by adjusting the Berry-Index (BI) by a health value (hv). The Berry-Index is defined as $1-\overset{n}{\underset{i=0}{\sum }}s_{i}^{2}$, where $s_{i}$ is the share of food, i in the total amount of energy intake and n is the total number of food items consumed [40]. First, the health value (hv) for each food code was derived by calculating a weighted average based on the number of equivalents of each food subgroup in a given food code. The health values were based on the Dietary Guidelines for Americans, 2015–2020 [13]. The resultant hv was then used to adjust the value of the calculated BI using the following equation $\left(1-\overset{n}{\underset{i=0}{\sum }}s_{i}^{2}\right)\mathrm{hv}$ from Drescher and colleagues [23]. A detailed description of HFBI can be found elsewhere [27]. The theoretical scores for HFBI can range between 0 and 1. The higher scores suggest not only even energy distribution from many food codes but also ensured that foods consumed were derived from healthful food groups. The consumption of less healthful foods or the daily energy contribution shifting to a relatively fewer number of food codes would result in lower HFBI scores.

The dissimilarity score was based on 10 attributes relevant to cardiovascular health. The attributes included were animal protein, plant protein, whole grains, refined grains, eicosapentaenoic acid (EPA) and docosahexaenoic acid (DHA), dietary fiber, sodium, alcohol, solid fats and oils. For each attribute, every food code was scored as either 0 or 1 using defined criteria. The food groups identified in the USDA’s Food Patterns and Equivalents database were assigned to each attribute group. The details of definitions and cutoff points for each attribute can be found elsewhere [27].

To estimate the dissimilarity score, Mahalanobis distance (MD) was used to derive the diversity of attributes of individual food consumption. The MD provided a standardized distance between two points in multivariate space, adjusting for the correlation among the variates [41]. This distance was defined here as $\sqrt{{\left(x_{i}-y_{i}\right)}^{T}\Sigma^{-1}\left(x_{i}-y_{i}\right)}$, where $x_{i}$ is the vector of attribute values for food x, $y_{i}$ is the vector of attribute values for food y, and Σ is the variance–covariance matrix among the attributes. Since all of the attributes included for this study were dichotomous, the covariance matrix was calculated using a method proposed by Karl Schweizer [42]. This method is based on the assumption that the observed dichotomous values are just indicators of a continuous normally distributed underlying latent construct. The theoretical values of MD could range from 0 to ∞. Larger values (≥3) are extremely unlikely, since MD essentially gives the number of standard deviations that a given food is away from the “attribute average”, and most foods will fall within one or two standard deviations from this average. Most of the calculated distances ranged between 0 and 1.5. A larger score suggests greater variety of food attribute values in a person’s daily diet consumption and vice versa.

### 2.5. Demographic and Health-Related Measures

Demographic variables included age (years), sex, race, income, education, and current cigarette smoking status. In the HANDLS study, sex was coded as female or male; race was self-reported as White or AA. Household income was self-reported either <125% poverty status or >125% poverty status as per the 2004 Health and Human Services poverty guidelines [28]. For this study, education was characterized as < high school education and ≥ high school education, and cigarette smoking was characterized as “Not a current smoker” and “Current smoker”. To interpret the results of statistical analysis, the reference categories for each of the abovementioned covariates include male, AA, <125% poverty status, ≥ high school education, and “current smoker”.

Weight was measured without shoes and coats using a calibrated Med-weigh model 2500 digital scale, and height was obtained with the subject’s heels and back against a height meter (Novel Products, Inc., Rockton, IL, USA). Body Mass Index (BMI), calculated from measured weight and height, was used to classify people as normal, overweight, or obese. In this study, normal weight was defined as BMI (kg/m^2^) from 18.5 to 24.9 kg/m^2^; overweight was defined as BMI from 25.0 to 29.9 kg/m^2^; obese was defined as BMI 30 kg/m^2^ or more. Underweight persons, defined as BMI < 18.5 kg/m^2^, were added to the normal weight category. There were 87, 62 and 47 underweight subjects at v1, v2 and v3, respectively.

### 2.6. Statistical Analyses

The statistical analyses included calculating means (±SE) for the three DD scores (count, evenness, and dissimilarity) for all three study visits. An analysis of Pearson’s product–moment correlation between each of the three DD scores was performed using IBM SPSS Statistics for Windows v25 (summary statistics) (2017; IBM Corporation, Armonk, NY, USA).

A logistic regression model was employed to assess the relationship between selected socio–demographic characteristics (age, sex, race, poverty status, and education) and having two days of dietary recall among participants who visited the MRV. The outcome was coded as “1” if a full recall was present and “0” if a full recall was not present.

To examine the change in DD scores over time, three linear mixed-effects models were used with DD measures as the dependent variables (separate model for each measure: count, evenness and dissimilarity). Random intercepts and random slopes for the time were incorporated into the models such that each participant’s starting value and trajectory over the course of the study were allowed to vary through the estimation of random effects. The model also adjusted for sex, race, poverty status, baseline age and education as fixed effects, and smoking and energy as time-dependent fixed effects. The baseline age and energy variables were mean-centered to facilitate model convergence and help keep parameter estimates on similar scales of magnitude. The formula for centered age and centered energy were as follows: (Age − 50)/10 and (Energy − 2000)/1000, respectively. Each model included years elapsed between visits (TIME) and 2-way interaction terms between TIME and key covariates; namely, race, sex, poverty status, education, baseline age, energy and smoking status. Those interaction terms are interpreted as the effects of those covariates on the slope or annual rate of change in the DD measure over time, independently of all other covariates. The main effects of covariates were also included in each model and are interpreted as fixed effects of covariates and outcomes on baseline (indicating which visit is baseline) DD measure, also independently of all other covariates. Conditional R^2^ values, which can be interpreted as the variance explained by both the fixed and random effects in a mixed model, were generated using a procedure described by Shinichi Nakagawa [43]. The degree to which the mixed model residuals and random effects conformed to a normal distribution were assessed using Q-Q plots. All normality assumptions were feasibly satisfied. The conditional variance for our mixed models were as follows: Count—0.500; Evenness—0.258; Dissimilarity—0.173. Repeated outcome measures averaged 1.8 visits per participant. We assumed the unavailability of outcomes to be missing at random. This analysis was performed using SAS v9.4 (2013, SAS Institute, Cary, NC, USA). The statistical significance for all analyses was established using *p* < 0.05.

## 3. Results

### 3.1. Population Characteristics

The demographic, health, and diet-related characteristics of the HANDLS study participants by study visit are presented in Table 1. The mean ages of the study participants who completed two dietary recalls at v1, v2, and v3 were 48 years, 53 years, and 57 years, respectively. At all three visits, the percentage of females was approximately 58%. Further, AAs appeared to represent more of the sample at v2 and v3 compared to v1 (61.4% and 60.9% vs. 57.9%, respectively). The percentage of participants with income <125% poverty status ranged from approximately 40–43%. More participants reported lower incomes at v1 compared to v2 and v3. Between 23 and 25% of participants had less than high school (<12th grade) education across study visits. The percentage of participants who reported being current smokers varied by visit, ranging from approximately 42 to 48% (Table 1). The mean BMI was 29.81 ± 0.17 kg/m^2^ at v1, 30.71 ± 0.17 kg/m^2^ at v2 and 30.94 ± 0.18 kg/m^2^ at v3 (Table 1).

A total of 2707 participants were recorded as having MRV visits. An accompanying logistic regression model revealed that, among these participants, younger age and having an income <125% of the poverty line status were significantly associated with increased odds of having two days of dietary recall as compared to older age and having an income >125% of the poverty line, respectively.

### 3.2. Dietary Diversity Scores and Energy Intakes

The medians of the count measures at v1, v2, and v3 were 0.429, 0.452, and 0.452, respectively. The medians of the evenness measures at v1, v2, and v3 were 0.120, 0.121, and 0.120, respectively. The medians of the dissimilarity measures at v1, v2, and v3 were 0.788, 0.758, and 0.809, respectively. For all three diversity scores, the means closely resembled the medians (Table 2).

The median energy intakes were 1829, 1905 and 1841 kcal at v1, v2 and v3, respectively. At all three visits, the mean energy intake was approximately 100 kcal higher than the median energy intake (Table 2).

### 3.3. Correlation Between DD Scores

The Pearson correlation values between the different measures of DD are presented in Table 3. The count scores were significantly positively correlated with evenness and dissimilarity scores at v1, v2 as well as at v3 (*p* < 0.01). The correlation between count and evenness was weaker than that found between count and dissimilarity. The correlation between evenness and dissimilarity scores was significant and negative at v2 (*p* < 0.01) as well as at v3 (*p* < 0.05).

### 3.4. Longitudinal Change in DD Scores

**Figure 2:**
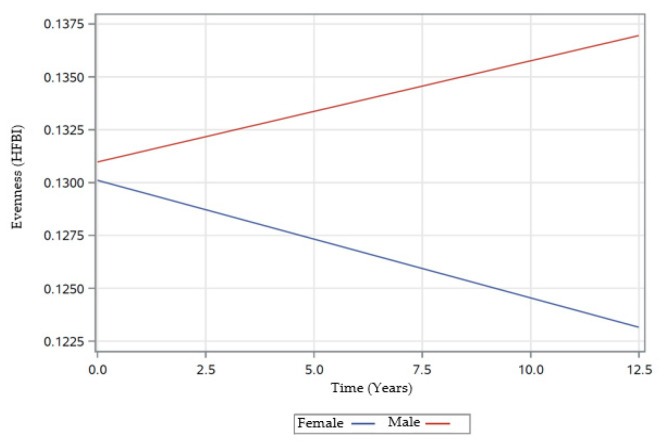
Rates of change in evenness scores over time by sex of participants examined in HANDLS study, 2004–2017. Abbreviations: HANDLS—Healthy Aging in Neighborhood of Diversity across the Life Span. HFBI—Healthy Factor Berry-Index based on health factor adjusted by Berry-Index using the formula by Drescher and colleagues [23].

**Figure 3:**
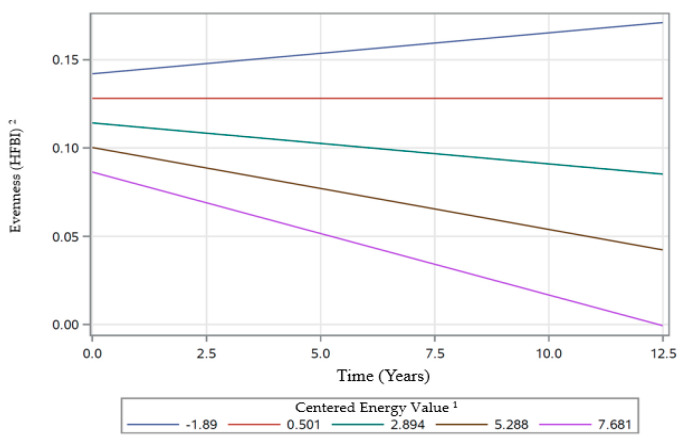
Rates of change in evenness scores over time by varying centered energy intake values of participants examined in HANDLS study, 2004–2017. Abbreviations: HANDLS—Healthy Aging in Neighborhood of Diversity across the Life Span. ^1^ Centered Energy calculated using formula: (Energy −2000)/1000). ^2^ HFBI—Healthy Factor Berry-Index based on health factor adjusted by Berry-Index using the formula by Drescher and colleagues [23].

**Figure 4:**
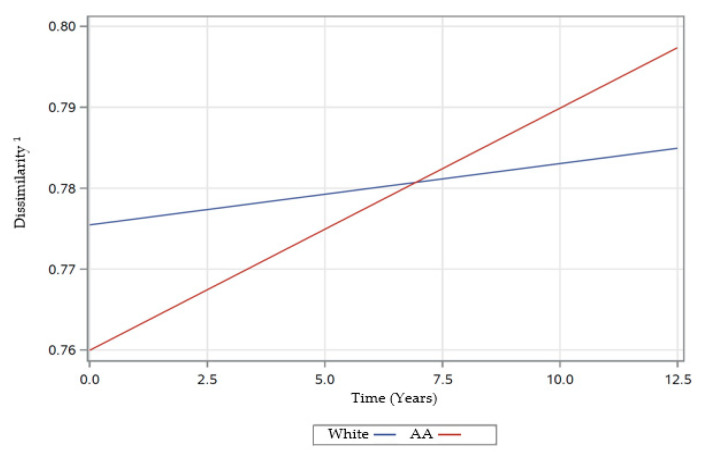
Rates of change in dissimilarity scores over time by race of participants examined in HANDLS study, 2004–2017. Abbreviations: AA—African–Americans, HANDLS—Healthy Aging in Neighborhood of Diversity across the Life Span. ^1^ Defined by Mahalanobis Distance [41].

**Figure 5:**
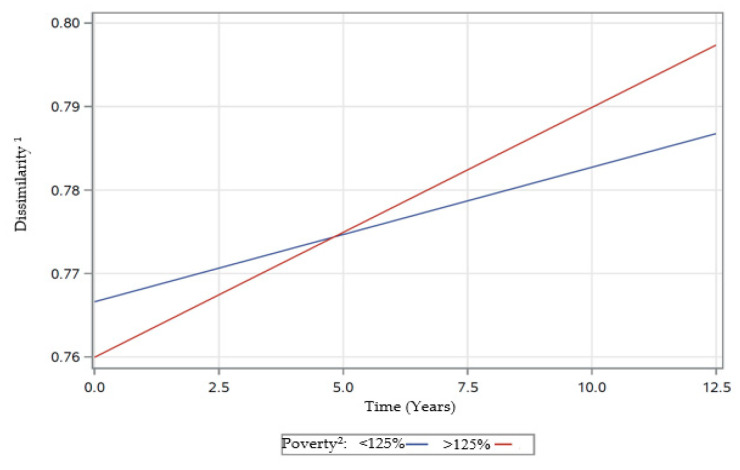
Rates of change in dissimilarity scores over time by poverty status of participants examined in HANDLS ^b^study, 2004–2017. Abbreviations: HANDLS—Healthy Aging in Neighborhood of Diversity across the Life Span. ^1^ Defined by Mahalanobis Distance [41]. ^2^ Poverty Status—Self-reported household income dichotomized into <125% and >125% of the 2004 Health and Human Services poverty guidelines [28].

**Figure 6:**
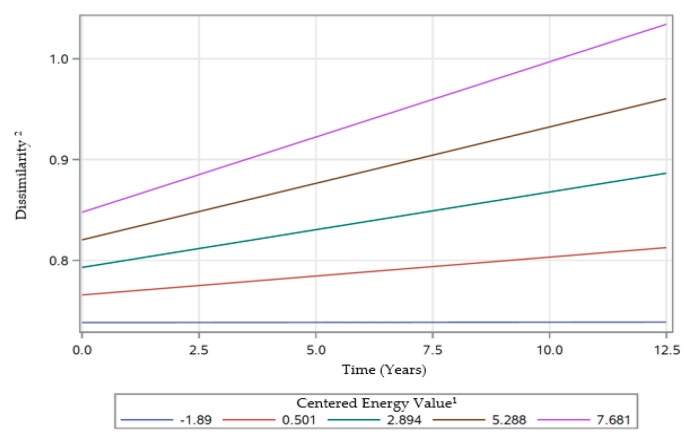
Rates of change in dissimilarity scores over time for varying centered energy intake values of participants examined in HANDLS ^c^study, 2004–2017. Abbreviations: HANDLS—Healthy Aging in Neighborhood of Diversity across the Life Span. ^1^ Centered Energy calculated using formula: (Energy−2000)/1000). ^2^ Defined by Mahalanobis Distance [41].

In the following three sections, Table 4, and Figure 2, Figure 3, Figure 4, Figure 5 and Figure 6 provide data regarding time changes in count, evenness, and dissimilarity.

#### 3.4.1. Linear Mixed-Effects Model Estimates for Count

There was a significant main effect of baseline age (β ± SE: 0.0052 ± 0.0021; *p* = 0.0131), sex (β ± SE: 0.0272 ± 0.0041; *p* < 0.0001) and race (β ± SE: 0.0082 ± 0.0039; *p* = 0.0377), indicating that overall, being older, female, or White was associated with a higher count score (Table 4). There was also a significant main effect of poverty status (β ± SE: 0.0163 ± 0.0041; *p* < 0.0001) indicating that those with income >125% poverty status had a higher count score compared to those with <125% poverty status. The significant effect of education (β ± SE: −0.0307 ± 0.0041; *p* < 0.0001) indicated that a participant with less than a high school education generally had lower count scores when compared to those with ≥ high school education. The significant main effect of energy (β ± SE: 0.0421 ± 0.0021; *p* < 0.0001) indicated that individuals who consumed greater caloric amounts also generally had higher count scores. Finally, the significant main effect of smoking (β ± SE: 0.0326 ± 0.0038; *p* < 0.0001) indicated that non-smokers generally had a higher count score than current smokers (Table 4). There was no significant interaction of time noted for any of the fixed effects included in the model; namely, baseline age, sex, race, poverty status, education, energy and smoking.

#### 3.4.2. Linear Mixed-Effects Model Estimates for Evenness

There were significant main effects for baseline age (β ± SE: 0.0055 ± 0.0012; *p* < 0.0001), and energy (β ± SE: −0.0058 ± 0.0012; *p* < 0.0001), indicating that overall, older individuals generally had higher evenness scores than younger individuals and individuals who consumed higher caloric amounts generally had lower evenness scores compared to their counterparts (Table 4). The significant main effect of smoking (β ± SE: −0.0150 ± 0.0023; *p* < 0.0001), indicated a lower evenness score in those who are not current smokers when compared to current smokers. Additionally, there were also significant two-way interactions of time with sex and energy. The interaction of time × sex (β ± SE: −0.0010 ± 0.0004; *p* = 0.0094), when coupled with the non-significant parameter estimates of time and indicated a decrease in the evenness score for females, while males experienced an increase in evenness score over time (Figure 2). The interaction of time × energy (β ± SE: −0.0010 ± 0.0002; *p* < 0.0001) indicated that individuals with high values of centered energy tended to experience a decrease in evenness over time, while individuals with low values of centered energy tended to experience an increase in evenness over time. The model tipping point between an estimated increase over time and an estimated decrease over time was at a centered energy value of approximately 0.5 (Figure 3).

#### 3.4.3. Linear Mixed-Effects Model Estimates for Dissimilarity

There was a significant main effect for baseline age (β ± SE: −0.0072 ± 0.0020; *p* = 0.0004), indicating that older individuals had lower dissimilarity scores than younger individuals (Table 4). Additionally, the significant main effect for sex (β ± SE: 0.0081 ± 0.0039; *p* = 0.0402) indicated that, regardless of visits, females generally had higher dissimilarity scores than males. The significant fixed effect for the race (β ± SE: 0.0155 ± 0.0038; *p* < 0.0001) indicated that Whites generally had higher dissimilarity scores than AAs. The positive fixed effect of energy (β ± SE: 0.0114 ± 0.0021; *p* < 0.0001) indicated that individuals consuming higher caloric amounts generally had higher dissimilarity scores than individuals consuming lower caloric amounts (Table 4).

There were also significant two-way interactions of time with race, poverty status, and energy (Table 4). The significant interaction of time × race (β ± SE: −0.0022 ± 0.0006; *p* = 0.0005), indicated that rates of change in dissimilarity differed over time by race. AAs experienced a greater increase in dissimilarity scores over time compared to Whites (Figure 4). The significant interaction of time × poverty status (β ± SE: −0.0014 ± 0.0006; *p* = 0.0325) indicated that rates of change in dissimilarity differed over time by poverty status (Table 4). Participants with lower income (< 125% poverty) experienced a greater increase in dissimilarity scores over time compared to those with higher income (> 125% poverty) (Figure 5). The significant interaction of time × energy (β ± SE: 0.0016 ± 0.0004; *p* < 0.0001) was indicative of different rates of change in dissimilarity over time by energy levels (Table 4). Participants with higher centered energy intakes experienced a greater increase in dissimilarity score over time, compared to those with lower centered energy intakes (Figure 6).

## 4. Discussion

This research is novel in that it used three different measures of DD simultaneously across three study visits in a sample of diverse urban US adults to examine the longitudinal change in DD measures. Our mixed-effects model estimates for count showed a significant main effect of age, sex, and race, indicating that overall, being older, female, or White was associated with a higher count score. To our knowledge, there was only one study that collectively used three similar different measures of DD. Otto and colleagues [20] evaluated the association of DD with abdominal obesity and type 2 diabetes in the Multi-Ethnic Study of Atherosclerosis. Their study sample was comprised of 5160 White, Hispanic, Black, and Chinese people aged 45–84 years old. They found higher count scores among Whites and females.

Otto and colleagues [20] reported higher evenness scores among Whites and females, which is in contrast to our findings. Our findings revealed a significant interaction of time × sex indicating a decrease in the evenness score for females over time. However, there was no significant effect of a race noted for evenness with the HANDLS study data.

Our mixed-effects model estimates for dissimilarity revealed a significant main effect for sex indicating higher scores among females. Additionally, our findings also showed a significant interaction of time × race indicating a greater increase in dissimilarity scores among AAs over time compared to Whites. Otto and colleagues [20] reported greater dissimilarity scores among Whites, and males compared to their counterparts, which is in contrast to our findings. The inconsistency in findings may be attributed to the difference in the formula used to derive the dissimilarity score, number, and definitions of selected food attributes, as well as outcome variables chosen. While Otto and colleagues [20] derived dissimilarity scores using Jaccard distance, we used Mahalanobis distance. Furthermore, their food attribute criteria were based on attribute tertiles of 100 g of food consumption, while we used the food’s nutrient composition per serving to define food attributes. Therefore, it is difficult to compare our findings with their results. To be able to compare findings across studies, differences between different DD measures need to be investigated and standardized.

Our finding provides evidence of higher count scores among Whites compared to their counterparts. A study by Kant and colleagues [44] also reported higher food group count scores as well as food group serving scores among Whites compared to Blacks. A higher percentage of Whites with high DD scores compared with non-Whites has also been reported in a cohort study based on a national probability sample of the US population that examined the association of DD to all-cause mortality [45]. Being female, higher income and education levels have been reported to be positively associated with DD scores based on the count [46], which corroborate with our findings.

In our study, evenness was calculated using BI as well as BI-adjusted with health factors. For all three study visits, HFBI evenness scores were lower than the unadjusted BI scores. Drescher and colleagues [23] reported similar findings consistent with our results despite the use of different health factors. The median HFBI score was 0.12 at each study visit. Compared to the theoretical range between 0 and 1, the score for our study sample suggests a more skewed pattern of energy distribution leaning towards the consumption of less healthful foods. High HFBI scores suggest not only even energy distribution from many food codes but also the consumption of foods from healthful groups.

The longitudinal analyses of our data across three study visits revealed significant changes in DD over time. The mixed-effects models estimate for each DD measure as an outcome variable presented different main effects and interactions of time with covariates. Our findings of a decline in evenness (HFBI) among females over time was consistent with those reported by Otsuka and colleagues [24] despite the use of a different DD measure. Otsuka and colleagues [24] used a diversity score based on Quantitative Index for Dietary Diversity using 13 food groups from 3-day dietary records. While they calculated the Quantitative Index for Dietary Diversity from the proportion of food intake that contributes to total energy to determine diversity, we used BI-adjusted health factor evenness scores.

Our study provided evidence of an increase in evenness scores by age, which was consistent with those reported by Otsuka and colleagues [24]. However, our findings presented no significant interaction of age and time over 13 years with any DD scores which is in contrast to those findings reported by Otsuka and colleagues [24]. They reported significant interactions of age and time with dietary diversity scores (*p* < 0.05) among community-dwelling Japanese adults over a 12-year longitudinal study. The possible explanation for this inconsistency could be attributed to the use of different measures and scoring methods to evaluate DD as well as to differences in the study population, dietary assessment tools, number of food groups and their subgroups. To be able to compare findings across studies, methods need to be standardized.

The mixed-effects model estimates with a dissimilarity score as the outcome revealed significant two-way interactions of time with race and poverty status, indicating a greater increase in dissimilarity among AAs and those with self-reported income <125% poverty status over time compared to their counterparts. The reason for such unexpected findings remains unclear and could be attributed to change in dietary patterns, shifting to the consumption of food with varied food attributes or skewing of energy distribution leaning towards the consumption of energy-dense, nutrient-poor foods. Previous findings from HANDLS study data showed that a western diet pattern was consumed among the study sample [47]. Additionally, previous findings from HANDLS data on counts of major food group consumption indicated racial differences, revealing a higher count of total protein food group among AAs, while Whites had the higher counts for the dairy group. We were unable to compare our findings with data from other researchers due to a lack of longitudinal studies evaluating the roles of race and income on dissimilarity measure.

The two-way interactions of time with energy were noted only for evenness and dissimilarity. As one’s caloric intake increased, the evenness scores tended to decrease over time, while their dissimilarity tended to increase over time. As mentioned earlier, the decrease in evenness would reflect lower variety with respect to the energy distribution of healthful foods. Higher dissimilarity in this study suggests the consumption of a greater variety of foods with attributes associated with a lower risk for developing cardiovascular disease.

One strength of this study was the racially and socio–economically diverse sample, a group underrepresented in the literature. Another strength was the use of three different measures of DD—namely count, evenness, and dissimilarity, which were based on two non-consecutive days of 24-h dietary recalls at three time points. The 24-h dietary recalls provide detailed information on foods and beverages consumed compared to other dietary assessment methods, such as the food frequency questionnaire. Additionally, the use of USDA AMPM in collecting dietary intake data reduces bias in the collection of energy intakes [33,48]. This study used Mahalanobis distance to derive dissimilarity scores. The advantage of using Mahalanobis distance is its ability to account for the correlation between attributes, thus eliminating the double-counting of attributes. Lastly, all three DD measures were based on healthful foods.

Despite the strengths of this study, there were some study limitations that deserve attention. Although 24-h dietary recalls collected with the USDA AMPM enabled us to capture accurate detail about foods consumed, the inherent errors associated with the method, as well as social desirability, may affect the reporting of intake behaviors [49]. Furthermore, our data analyses were limited to the urban population residing in the Baltimore, Maryland area. Therefore, the results might be different if the same analyses were conducted with a nationally representative data set, such as NHANES.

## 5. Conclusions

The study provided a unique insight into changes in aspects of DD across adulthood. Dietary diversity, based on dissimilarity, revealed a significant change by race and income over time. However, the lack of longitudinal studies that have used dissimilarity measures to evaluate socio–demographic differences over time limits the comparability of studies. To enable greater insight into the practical application of aspects of DD and their role in maintaining a healthful diet, future research first needs to focus on the development and validation of standard measures of DD.

## Figures and Tables

**Table 1:** Sociodemographic and health characteristics of individuals examined in HANDLS study, 2004–2017.

Characteristics	Study Visits		
Mean ± SE or %	Visit 1 *N* = 2177	Visit 2 *N* = 2140	Visit 3 *N* = 2066
**Demographics**			
Age, years	48.33 ± 0.20	53.20 ± 0.19	56.63 ± 0.20
Female, %	56.5	58.8	59.0
African–American, %	57.9	61.4	60.9
Poverty Status, <125%	42.9	39.8	40.8
Education, % < High School	25.3	22.9 (*n* = 2133)	23.4 (*n* = 2052)
Cigarette Smoker, %	48.3 (*n* = 2004)	41.8 (*n* = 1903)	46.5 (*n* = 1808)
**Health Conditions**			
BMI, kg/m^2^	29.81 ± 0.17 (*n*= 2174)	30.71 ± 0.17 (*n* = 2136)	30.94 ± 0.18 (*n* = 2048)

Abbreviations: BMI—Body Mass Index, HANDLS—Healthy Aging in Neighborhoods of Diversity across the Life Span.

**Table 2:** Dietary diversity scores and energy intakes of individuals examined in HANDLS study, 2004–2017.

Characteristics	Study Visits					
	Visit 1 (*N* = 2177)		Visit 2 (*N* = 2140)		Visit 3 (*N* = 2066)	
Diet-Related Measures	Median	(Mean ± SE)	Median	(Mean ± SE)	Median	(Mean ± SE)
Count Score ^1^	0.429	0.430 ± 0.002	0.452	0.449 ± 0.002	0.452	0.445 ± 0.002
Evenness Score (HFBI) ^2^	0.120	0.129 ± 0.001	0.121	0.131 ± 0.001	0.120	0.128 ± 0.001
Dissimilarity Score ^3^	0.788	0.785 ± 0.002	0.758	0.751 ± 0.002	0.809	0.802 ± 0.002
Energy, kcal	1829	2006 ± 21	1905	2025 ± 18	1841	1999 ± 11

Abbreviations: HANDLS—Healthy Aging in Neighborhoods of Diversity across the Life Span. ^1^ Based on the consumption of at least one-half of serving equivalent for 21 food groups. ^2^ HFBI—Healthy Factor Berry-Index based on health factor adjusted by Berry-Index using the formula by Drescher and colleagues [23]. ^3^ Defined by Mahalanobis Distance [41].

**Table 3:** Correlation between dietary diversity measures across study visits for HANDLS study participants, 2004–2017.

	Count	Evenness (HFBI)	Dissimilarity
**Dietary Diversity Measures, visit 1 (*N* = 2177)**			
Count ^1^	−		
Evenness (HFBI) ^2^	0.169 **	−	
Dissimilarity ^3^	0.200 **	−0.020	−
**Dietary Diversity Measures, visit 2 (*N* = 2140)**			
Count ^1^	−		
Evenness (HFBI) ^2^	0.166 **	−	
Dissimilarity ^3^	0.219 **	−0.079 **	−
**Dietary Diversity Measures, visit 3 (*N* = 2066)**			
Count ^1^	−		
Evenness (HFBI) ^2^	0.167 **	−	
Dissimilarity ^3^	0.209 **	−0.043 *	−

Abbreviations: HANDLS—Healthy Aging in Neighborhoods of Diversity across the Life Span. ^1^ Based on the consumption of at least one-half of serving equivalent for 21 food groups. ^2^ HFBI—Healthy Factor Berry-Index based on health factor adjusted by Berry-Index using the formula by Drescher and colleagues [23]. ^3^ Defined by Mahalanobis Distance [41]. ** Correlation is significant at *p*-value < 0.01 level. * Correlation is significant at *p*-value < 0.05 level.

**Table 4:** Predictors of Count, Evenness, and Dissimilarity over time: linear mixed-effects regression models, HANDLS study, 2004–2017.

Variables	Count ^1^			Evenness (HFBI) ^2^			Dissimilarity ^3^		
	Estimate	SE	*p*	Estimate	SE	*p*	Estimate	SE	*p*
Intercept	0.3963	0.0047	<0.0001 ***	0.1386	0.0029	<0.0001 ***	0.7605	0.0048	<0.0001 ***
Time (Years)	0.0010	0.0007	0.1639	0.0002	0.0005	0.6345	0.0032	0.0008	<0.0001 ***
BLAge ^4^	0.0052	0.0021	0.0131 *	0.0055	0.0012	<0.0001 ***	−0.0072	0.0020	0.0004 ***
Time*BLAge	0.0005	0.0003	0.1688	−0.0003	0.0002	0.1675	0.0004	0.0003	0.2895
Sex (Female)	0.0272	0.0041	<0.0001 ***	−0.0009	0.0024	0.7174	0.0081	0.0039	0.0402 *
Time*Sex (Female)	0.0005	0.0007	0.4295	−0.0010	0.0004	0.0094 **	−0.0006	0.0007	0.3932
Race (White)	0.0082	0.0039	0.0377 *	−0.0013	0.0023	0.5732	0.0155	0.0038	<0.0001 ***
Time*Race (White)	−0.0010	0.0006	0.2532	0.0002	0.0004	0.6110	−0.0022	0.0006	0.0005 ***
Poverty Status (> 125% Poverty)^5^	0.0163	0.0041	<0.0001 ***	0.0011	0.0024	0.6564	0.0067	0.0039	0.0914
Time*Poverty Status (> 125% Poverty)	0.0000	0.0006	0.9493	−0.0004	0.0004	0.3568	−0.0014	0.0006	0.0325 *
Education (< High school)	−0.0307	0.0041	<0.0001 ***	−0.0041	0.0025	0.0958	0.0075	0.0041	0.0684
Time*Education (< High school)	0.0009	0.0007	0.2193	0.0004	0.0004	0.3867	0.0001	0.0007	0.8757
Smoking (Not A Current Smoker)	0.0326	0.0038	<0.0001 ***	−0.0150	0.0023	<0.0001 ***	−0.0044	0.0038	0.2463
Time*Smoking (Not A Current Smoker)	−0.0001	0.0006	0.8721	0.0005	0.0004	0.2054	−0.0003	0.0006	0.6402
CenEng ^6^	0.0421	0.0021	<0.0001 ***	−0.0058	0.0012	<0.0001 ***	0.0114	0.0021	<0.0001 ***
Time*CenEng	0.0005	0.0004	0.2027	−0.0010	0.0002	<0.0001 ***	0.0016	0.0004	<0.0001 ***

Abbreviations: HANDLS—Healthy Aging in Neighborhood of Diversity across the Life Span. ^1^ Based on the consumption of at least one-half of serving equivalent for 21 food groups. ^2^ HFBI—Healthy Factor Berry-Index based on health factor adjusted by Berry-Index using the formula by Drescher and colleagues [23]. ^3^ Dissimilarity—Defined by Mahalanobis Distance [41]. ^4^ BLAge—Baseline Age = (Age–50/10). ^5^ Poverty Status—Self-reported household income dichotomized into <125% and >125% of the 2004 Health and Human Services poverty guidelines [28]. ^6^ CenEng—Centered Energy = (Energy−2000)/1000). *p-values significant at < 0.05. ** *p*-value significant at < 0.01. *** *p*-value significant at < 0.001.
